# Type Ib endoleak repaired with a thoracic endovascular graft inside previous visceral debranching bypass in a patient with chronic type B aortic dissection

**DOI:** 10.1016/j.jvscit.2023.101186

**Published:** 2023-04-12

**Authors:** Cristina Tello-Díaz, Jose Maria Romero Carro, Begoña Soto Carricas, Jorge Moreno, Carmen Aloy Ortiz, Jaume F. Dilmé

**Affiliations:** Angiology, Vascular Biology and Inflammation Laboratory, and Department of Vascular and Endovascular Surgery, Sant Pau Institute of Biomedical Research, Universitat Autónoma de Barcelona, Barcelona; and the CIBER de Enfermedades Cardiovasculares, Instituto de Salud Carlos III, Madrid, Spain

**Keywords:** Dissection, Endograft, Endovascular, Reintervention

## Abstract

We present the case of a patient with chronic type B aortic dissection with a previous iliac to visceral debranching graft and thoracoabdominal endograft who, because of a type Ib endoleak and aortic diameter enlargement, required a complex solution involving placement of a thoracic endovascular graft inside a Dacron graft with a 180° curved shape in three-stage surgery. At 9 months of follow-up, he had no evidence of type I endoleaks, and the aortic diameter had decreased.

Type B aortic dissection (TBAD) has an incidence of 2.9 to 4.0 per 100,000 person-years and the incidence has been increasing.^1^ Historically, 14 days after the acute event, TBAD is defined as “chronic.” However, currently, the preferred classification of TBAD is as follows: hyperacute, <24 hours; acute, 1 to 14 days; subacute, 15 to 90 days; and chronic, >90 days.^2^ One of the aims of treatment of uncomplicated TBADs is to prevent the progression and aneurysmal degeneration of the aorta. In the chronic phase, TBAD has been estimated to develop aneurysmal degeneration in >20% to 40% of patients.^3^

Medical treatment with antihypertensive drugs remains the cornerstone of treatment of chronic TBAD (cTBAD).^3^ Surgery is indicated when aortic dilatation >50 to 60 mm occurs or symptoms appear. Open surgery has been the treatment of choice for these patients; however, the advent of endovascular treatment has resulted in new treatment possibilities. To the best of our knowledge, no data are available regarding comparisons between open and endovascular repair; however, endografts have shown good results in the subacute and chronic phases.^4^

The management of cTBAD is complex, requiring hybrid solutions and, sometimes, multiple reinterventions. We describe the management of a patient with cTBAD, who had previously been treated with a visceral debranching graft and thoracoabdominal endograft. The patient presented with progressive enlargement of the aortic diameter due to a type Ib endoleak. Three-stage surgery was performed, which included placement of a thoracic endovascular graft inside the visceral debranching Dacron graft with a 180° curved shape.

## Case report

We report the case of a 67-year-old man with a history of smoking and hypertension who, at 45 years of age, had presented with an uncomplicated TBAD. The patient provided written informed consent for the report of his case details and imaging studies.

At the diagnosis, given the absence of complications and correct perfusion of the visceral arteries and lower extremities, he was treated conservatively in the coronary care unit with medical treatment of his hypertension and analgesia for pain control. His clinical course during admission was positive, and he was discharged with close follow-up with computed tomography angiography, scheduled every 6 months for the first year. After 1 year of stability, the imaging test was performed annually. During surveillance, a progressive increase in the aortic diameter was observed. The aortic diameter had increased from 50 mm to 73 mm in 1 year, and it was decided to proceed with surgical treatment.

The patient, who was 57 years old at the time, was in good physical condition, hypertension as a part of his medical history, and a thoracoabdominal aneurysm, Crawford type II. Although open surgery was considered, it was finally decided to perform a hybrid approach to minimize surgical invasiveness. First, a distal landing zone was created with visceral debranching graft, and next, endovascular treatment of the thoracic aorta was performed.

In the first stage, visceral debranching bypass was performed from the left common iliac artery to the right renal artery, superior mesenteric artery, and hepatic artery (the left renal artery was atrophic) with a 16 × 8-mm Dacron graft (Maquet, Rastatt, Germany). In the second stage, the descending thoracic and visceral abdominal aorta was covered with Valiant 34 × 34 × 100-mm and 34 × 34 × 150-mm endografts (Medtronic Vascular, Santa Rosa, CA).

One month later, because of a distal endoleak of the previous endograft and an isolated right common iliac dissection, we placed a Valiant 34 × 34 × 100-mm endograft (Medtronic Vascular) in the abdominal aorta and a Talent 18 × 12 × 80-mm stent graft (Medtronic Vascular) in the right common iliac artery.

**Fig 1:**
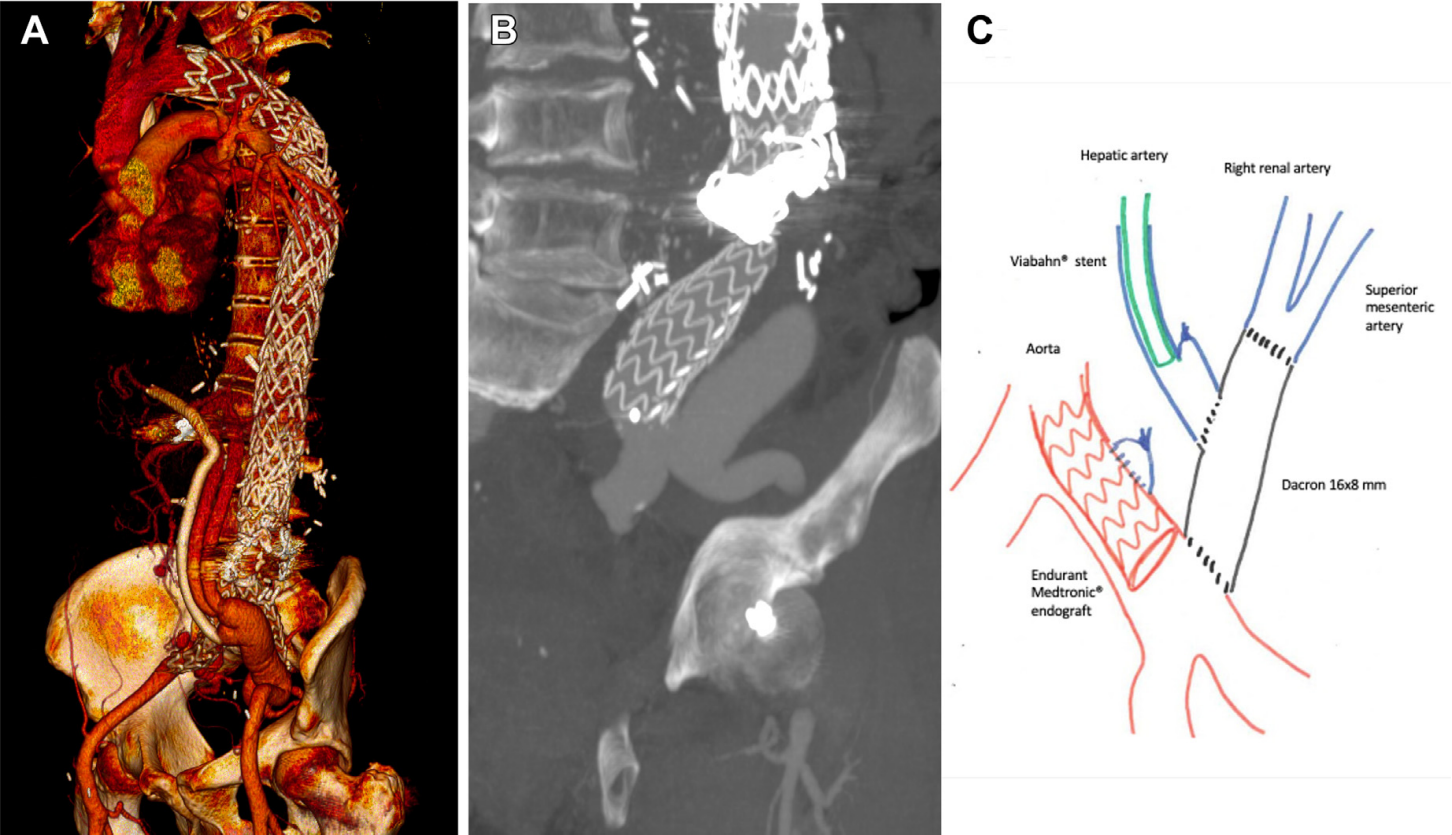
**A,** Three-dimensional computed tomography reconstruction showing hybrid treatment of the type B aortic dissection (TBAD) with complete endograft coverage of the descending thoracic and abdominal aortas and visceral debranching, including salvage of one of the debranching branches with a stent. **B,** Computed tomography reconstruction showing in detail the visceral debranching bypass from the external iliac artery and the endograft ending in the common iliac artery, at the edge of the visceral bypass. **C,** Diagram illustrating mobilization of the visceral debranching bypass from the common iliac artery to the external iliac artery to cover the common iliac artery with the endograft. Also shown is stenting of the iliohepatic bypass after thrombectomy.

Two years after the last surgery, in 2013, progressive aneurysmal degeneration was noted, due to a distal endoleak. First, the proximal anastomosis of the visceral debranching bypass was redone distally at the iliac bifurcation to cover the left common iliac artery. Next, we placed bifurcated Endurant II 36 × 16 × 166-mm and 16 × 24 × 124-mm endografts (Medtronic Vascular) overlapped with the previous endograft to both iliac bifurcations. During this procedure, the bypass branch to the hepatic artery thrombosed and required thrombectomy and stenting (Fig 1). After this surgery, the patient suffered splenic bleeding with hypovolemic shock that required splenectomy and multiple transfusions. His postoperative course was complicated by coagulopathy, acute respiratory failure, and infection of the abdominal hematoma requiring a long admission in the intensive care unit. His recovery was slow but satisfactory.

Despite the previous interventions, in 2014, 1 year after the last intervention, type II and III endoleaks were detected. The inferior mesenteric artery was embolized with Barricade coils (Blockade Medical, Irvine, CA), and three new thoracic stent grafts were placed from the distal edge of the left subclavian artery to the previous bifurcated endograft (Valiant 36 × 36 × 150 mm, 34 × 34 × 100 mm, and 34 × 34 × 150 mm; Medtronic Vascular).

**Fig 2:**
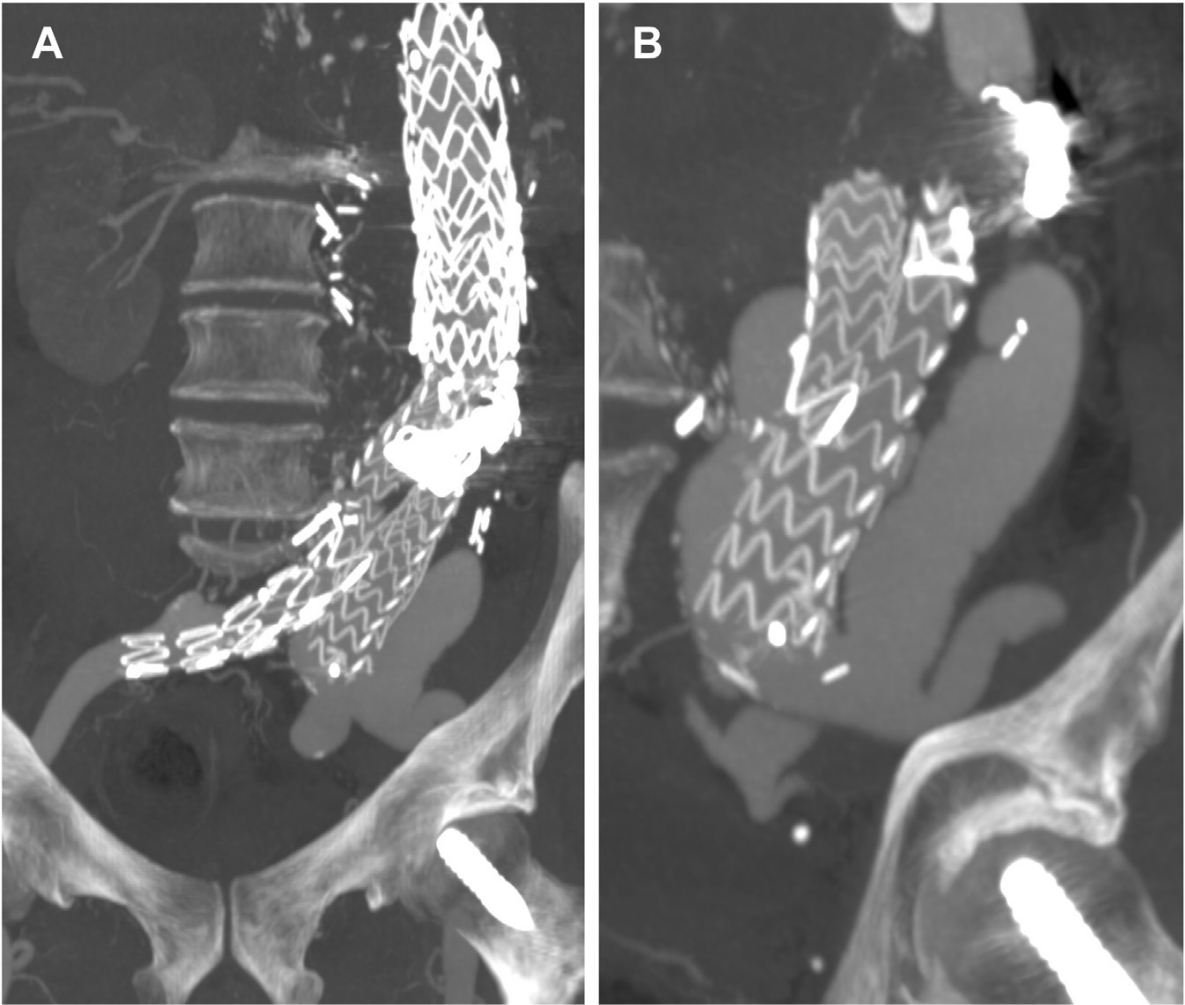
Computed tomography reconstructions evidencing type Ib endoleak through both common iliac arteries. **A,** Anteroposterior projection. **B,** Lateral projection showing the relationship with visceral debranching.

After the last surgery, the subsequent computed tomography scans showed stability. However, in 2021, 7 years later, a type Ib endoleak from both iliac arteries and an increase in the aortic diameter to 109 mm were detected (Fig 2). At that time, 10 years after the first intervention, our patient was 67 years old. Total reconversion to open surgery was not considered because of the multiple reinterventions, hostile abdomen, and high risk of visceral bypass complications. In addition, the patient was reluctant to undergo another open surgery.

**Fig 3:**
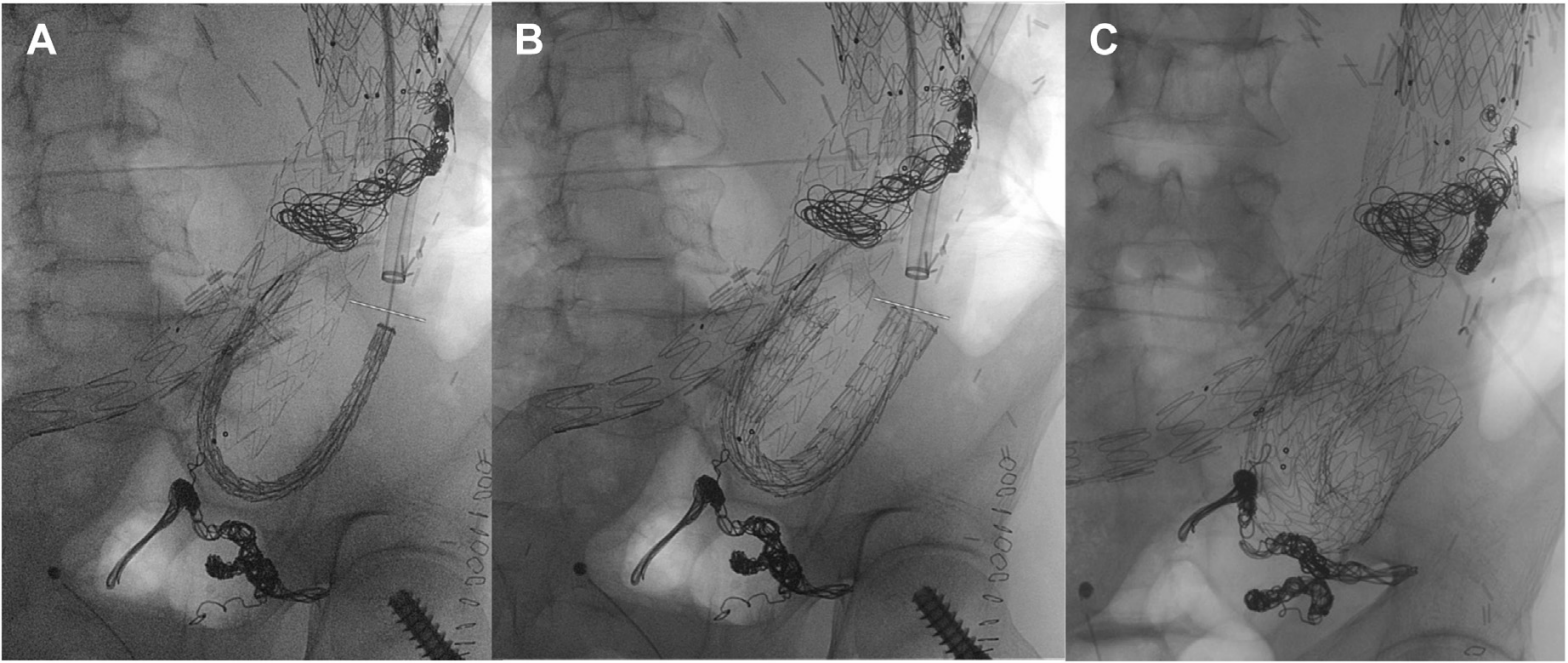
Fluoroscopy images showing placement of the cTAG endograft from the common iliac artery to the visceral debranching graft, acquiring a 180° curved shape. **A,** Placement inside the visceral debranching. **B,** Image after device release. **C,** Image after balloon dilation of the prosthesis.

We planned a three-stage surgery to repair the type Ib endoleak. First, through a right femoral percutaneous access, the left hypogastric artery was embolized with coils (Ruby System; Penumbra, Alameda, CA). Second, a 13 × 50-mm Viabahn stent graft (W.L. Gore & Associates, Flagstaff, AZ) was deployed in the right external iliac artery to cover the right hypogastric artery, and femorofemoral bypass (right to left) with an 8-mmpolytetrafluoroethylene graft (W.L. Gore & Associates) was performed. Finally, through a left retroperitoneal approach, a 10-mm Dacron graft (Maquet) was sutured to the previous visceral debranching (right renal artery branch) for endovascular cannulation. Through this Dacron conduit and right brachial artery access, a “through and through” was performed, allowing us to place a 20F, 65-cm DrySeal Flex sheath (W.L. Gore & Associates) in the aorta across the previous debranching. A 28 × 28 × 150 cTAG conformable endograft (W.L. Gore & Associates) was placed from the left common iliac artery to the visceral debranching graft, leaving the thoracic stent graft with a 180° curve (Fig 3). Completion arteriography revealed correct deployment of the endograft, with a small residual type Ib endoleak. The Dacron conduit was removed without incident. The postoperative course was uneventful with patent femorofemoral and visceral bypasses. The patient was discharged on the fourth postoperative day.

**Fig 4:**
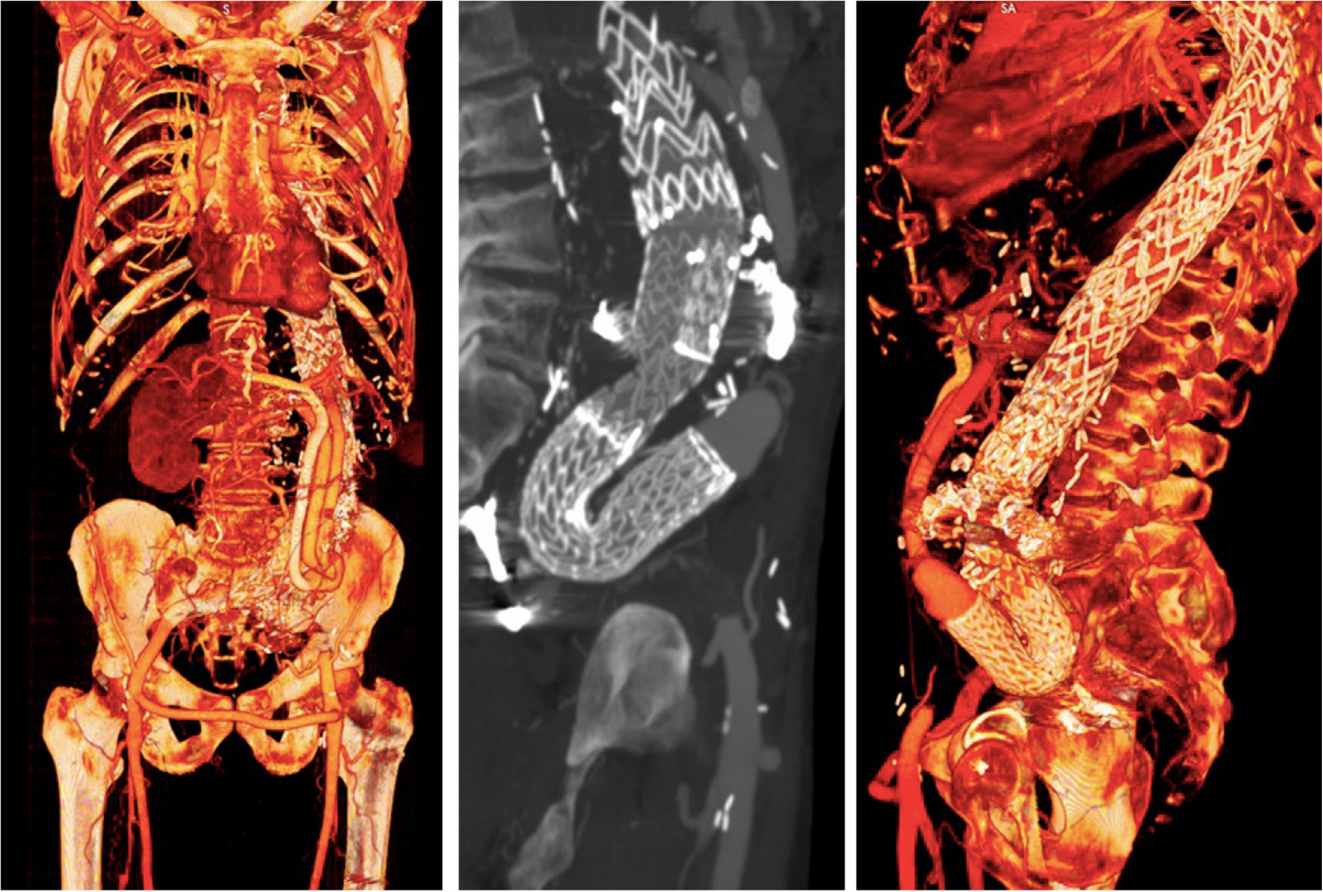
Computed tomography reconstructions showing endograft placement, with a 180° curved shape within the visceral debranching Dacron graft without kinking or endoleaks.

One year after surgery, the patient was doing well and was able to resume his daily activities without any limitations. A 9-month follow-up computed tomography angiography scan demonstrated no evidence of type I endoleaks (Fig 4), patency of the visceral debranching and thoracic endovascular graft with a 180° curved shape, a decreased diameter of the right iliac artery, and stability of the aortic diameter.

## Discussion

We present the complex repair of a type Ib endoleak in a patient with multiple interventions for cTBAD. The type Ib endoleak was treated with a hybrid solution that included off-label placement of a thoracic endovascular graft inside a visceral debranching graft.

The management of cTBAD remains controversial. The INSTEAD (investigation of stent grafts in patients with type B aortic dissection) trial revealed no advantages of thoracic endovascular repair (TEVAR) compared with medical therapy at 2 years.^4^ Surgical treatment is justified when aneurysmal degeneration (30%),^5^ rapid aortic growth, or symptoms are detected during follow-up. cTBAD with a descending thoracic aortic diameter >55 mm should be considered for treatment owing to the increased ruptured risk.^3^

Open surgery has provided good results but with high morbidity.^6^^,^^7^ Hybrid approaches (visceral debranching and aortic endografting) allow for treatment of TBADs, avoiding extensive aortic exposure and total clamping of the aorta and allowing for circulatory support. Thus, they are considered less invasive, although they could also be indicated for patients with low comorbidities.^6^

At present, open surgery is being replaced by endovascular treatment, even without data from randomized studies.^8^ Good early and long-term outcomes have been reported for endovascular strategies to treat cTBAD, especially for unfit patients.^9^^,^^10^ In 2013, Nienaber et al^8^ reported the benefits in terms of survival of preemptive TEVAR, in addition to medical treatment, for patients with TBAD.

Endovascular treatment aims to prevent persistent perfusion of the false lumen to obtain a reduction in the aortic diameter.^1^^,^^11^ TEVAR in the chronic phase of TBAD can be challenging and often requires extensive aortic coverage.^3^^,^^11^^,^^12^ The flap stiffness in this chronic stage and the lack of remodeling implies that reinterventions will be frequent (18%-50%).^13^^,^^14^

Often, using TEVAR alone will not be enough to achieve aortic remodeling owing to persistent false lumen filling. Many adjuvant techniques to TEVAR have been described, most of which are off label.^15^ The aim could be to occlude the false lumen, such as the Knickerbocker and candy plug techniques.^16^, ^17^, ^18^ In addition, new fenestrations have been performed in the false lumen to complete an endovascular treatment.^19^ Thus, for cTBAD, some novel techniques have been used to increase the benefits of this endovascular approach.

We have described the case of a patient with cTBAD, who had initially been treated with a hybrid approach owing to his good physical condition but who had undergone multiple reinterventions because of the development of distal endoleaks filling the false lumen. When he presented yet again with an endoleak but was considered at high risk of multiple complications owing to the hostile abdomen, complete conversion to open surgery was not considered. We proposed an endovascular solution with placement of a thoracic endograft inside a Dacron graft, with a 180° curve, pushing the capabilities of the endovascular grafts to the limit. We chose the cTAG conformable endograft (W.L. Gore & Associates) because of its great flexibility. The thoracic graft showed great adaptation to the shape of the Dacron graft without kinking.

## Conclusions

We present the case of a patient with cTBAD who had required multiple reinterventions. He underwent treatment of a type Ib endoleak using a thoracic endograft inside a visceral debranching graft, adapting it to a 180° angle.

This case confirms the difficult management of cTBAD and reflects the usual high rate of reinterventions. Nevertheless, we have demonstrated creative endovascular possibilities for solving the complicated cases of our patients.
